# Inequities in Healthcare utilization: results of the Brazilian National Health Survey, 2013

**DOI:** 10.1186/s12939-016-0444-3

**Published:** 2016-11-17

**Authors:** Cristiano Siqueira Boccolini, Paulo Roberto Borges de Souza Junior

**Affiliations:** 1 Institute of Scientific and Technological Communication and Information in Health, Oswaldo Cruz Foundation, Rio de Janeiro, Brazil; 2 Av. Brasil, 4.365 - Pavilhão Haity Moussatché - Manguinhos, Rio de Janeiro, CEP: 21040-900 Brazil

**Keywords:** Health equity, Equity in access, Health services accessibility, Primary Health Care, Unified Health System

## Abstract

**Background:**

The Brazilian Unified Health System is a public healthcare system that has universal and equitable access among its main principles, but the continental size of the country and the complexity of the public health system complicate the task of providing equal access to all. We aim to investigate the factors associated with inequities in healthcare utilization in Brazil.

**Methods:**

We employed data from a nationally representative cross-sectional study (2013 National Health Survey; *n* = 60,202). The outcome was underutilization of healthcare by adults, defined as lack of utilization of one or more of these services: physician or dentist consultation, and blood glucose or blood pressure screening. A logistic regression model, considering the complex sample, was employed (alpha = 5 %).

**Results:**

0.7 % of the sample never visited a physician, 3.3 % never visited a dentist, 3 % never underwent blood pressure screening, 11.5 % never underwent blood glucose screening, and 15 % never utilized at least one of these services. Multivariate models showed a higher likelihood of underutilization of healthcare among individuals of the lowest social class “E” (AOR = 6.31, 95 % CI = 3.76–10.61), younger adults (Adjusted Odds Ratio, or AOR = 4.40, 95 % CI = 3.78–5.12), those with no formal education or incomplete primary education (AOR = 2.93, 95 % CI = 2.30–3.74), males (AOR = 2.16, 95 % CI = 1.99–2.35), and those without private health insurance (AOR = 2.11, 95 % CI = 1.83–2.44). Individuals self-classified as “white” were less likely to report underutilization (AOR = 0.82, 95 % CI = 0.75–0.90).

**Conclusions:**

Despite recent expansion of primary healthcare and oral health programs in Brazil, we observed gaps in healthcare utilization among the most vulnerable segments of the population.

## Background

The Brazilian constitutional right to healthcare is addressed through the Unified Health System (*Sistema Único de Saúde* or SUS, in Portuguese), which is guided by the principles of universal and equitable access to healthcare. These principles were fundamental to an extensive health system reorganization driven by civil society soon after the adoption of the 1988 constitution [1]. Brazil is a large country marked by persistent regional and social inequities in health [2]. Although healthcare utilization has become more equitable from 1998 to 2008, mostly due to improvements in primary healthcare, unequal access persists, with the rich having higher utilization than the poor [3].

The major public health problems in Brazil are related to Non-Communicable Diseases (NCDs), responsible for 72 % of all deaths in 2007. Even with a historical reduction of NCD mortality, the highest rates of mortality and morbidity for these diseases are concentrated among the poorest [4], revealing persistent inequity in healthcare access [1]. According to 2013′s National Health Survey (*Pesquisa Nacional de Saúde* or PNS), Brazil’s most prevalent self-reported diseases among adults were hypertension, depression, arthritis, and diabetes mellitus (corresponding to 21.4, 7.6, 6.4 and 6.2 %, respectively) [5]. The leading causes of lost disability-adjusted life years (DALYs) in 2008 were cardiovascular disease, mental disorders, and diabetes, which, together with the other NCDs, were responsible for 77.2 % of all lost Brazilian DALYs [6].

Analyzing healthcare utilization among adults in Brazil, the general scenario seems adequate for those that reported being diabetic [7] or having high blood pressure [8], according to the 2013 PNS. However, 11.5 and 3 % of the population were never screened for blood glucose [7] and blood pressure, respectively [8]. Another obstacle in Brazilian public health is the lack of access to oral healthcare. Although access has expanded over the last decades, there are persistent income inequalities in the distribution of services [9].

Although healthcare utilization for those reporting NCDs (like diabetes and hypertension) seems adequate, barriers to medical treatment and diagnosis for these diseases, and for oral health prevention and treatment, are still an issue for many in the Brazilian population. We aim to investigate the factors associated with inequities in healthcare underutilization in Brazil. Our hypothesis is that the poorest and most vulnerable underutilize the healthcare system.

## Methods

### Study design and sampling

This is a cross-sectional study using data from the 2013 PNS, a survey representative of the Brazilian population. The PNS sample was a subsample of the Master Sample of the Integrated Household Surveys System of the Brazilian Institute of Geography and Statistics. Three-stage cluster sampling was adopted. The primary sampling unit (PSU) was comprised of sectors of the Census and selected by simple random sampling, maintaining the stratification of PSUs used in the Master Sample. The secondary unit was the household and the third unit was a random sample of residents over the age of 18, from each household. The minimum size for the sample was 1,800 households per Brazilian state. Initially, households were selected. A total of 60,202 respondents from 81,167 selected households were interviewed. The non-response rate was 8.1 %. Interviews were carried out over the period August 2013 to February 2014. Details regarding the process of sampling and weighting are available in a prior publication [10].

### Outcome and associated variables

This study considered four questions related to the outcome. The first two inquired about physician and dentist visits: “When was the last time you visited a physician?” and “When was the last time you visited a dentist?” The remaining questions inquired about blood pressure and blood glucose measurement: “When was the last time you had your blood pressure checked?” and “When was the last time you had your blood glucose/sugar checked?” Each of these questions had six answer categories: “less than 6 months,” “more than 6 months and less than 1 year,” “more than a year and less than 2 years,” “more than 2 years and less than 3 years,” “more than 3 years,” and “never.” The outcome “underutilization of the health system” was defined when the subject answered positively to one or more of the following: never went to a physician, never went to a dentist, never had blood pressure checked, or never had blood glucose checked.

We also calculated a socioeconomic index, using variables indicative of household assets, education of the head of the household, and the presence of a paid housekeeper. Based on these parameters we used a previously established method to classify the population into five socioeconomic categories: A and B (upper classes); C (middle class); and D and E (lower classes) [11].

### Statistical analysis

Initially, a description of the prevalence of each variable comprising the outcome “underutilization” was carried out, with 95 % confidence intervals (CI). Then, adjusted prevalence rates of underutilization were estimated using logistic regression. The models were adjusted for the following variables: sex, race (divided into white and non-white), educational level (no schooling, primary incomplete, secondary incomplete, undergraduate incomplete, undergraduate complete or higher); age groups (18–29, 30–39, 40–49, 50–59, 60 or more), Brazilian socioeconomic criteria (classes A to E), private health insurance (yes or no), and by Family Healthcare Strategy coverage (yes or no). The Odds Ratio (OR) and 95 % confidence interval were estimated for each variable. Finally, a logistic regression model was used to adjust the prevalence of the outcome “underutilization” for sex, educational level, age, race, and Family Healthcare Strategy coverage.

## Results

Among the 60,202 individuals included in the PNS, 18 % were aged 60-years or older, nearly 40 % did not attend school at all or did not complete primary school. Regarding socioeconomic classification, approximately 25 % of respondents were in classes D or E (corresponding to the lower classes) and 70 % had no private health insurance (Table 1).

**Table 1:** Subjects socioeconomic characteristics and health care coverage - Brazilian National Health Survey, 2013^a^

Variable/categories	Prevalence %^b^	(95 % CI)^c^	*n*
Sex			
Male	47.1	(46.4–47.9)	25920
Female	52.9	(52.1–53.6)	34282
Skin color^d^			
White	47.5	(46.7–48.3)	24106
Non-white	52.5	(51.7–53.3)	36096
Educational level			
No education/primary incomplete	38.9	(38.1–39.8)	24083
Secondary incomplete	15.5	(15.0–16.1)	9215
Undergraduate (college) incomplete	32.8	(32.1–33.5)	19149
Undergraduate complete and above	12.7	(12.0–13.5)	7755
Social Class			
E	2.8	(2.6–3.1)	2563
D	21.8	(21.1–22.5)	15610
C	43.0	(42.1–43.9)	25818
B	28.6	(27.7–29.5)	14514
A	3.8	(3.4–4.4)	1697
Age (years)			
>=18 and <30	26.1	(25.4–26.7)	14321
>=30 and <40	21.6	(21.0–22.2)	14269
>=40 and <50	18.1	(17.5–18.6)	11405
>=50 and <60	16.2	(15.7–16.7)	9030
>=60	18.0	(17.5–18.7)	11177
Private Health Insurance			
no^e^	69.7	(68.8–70.7)	43834
yes	30.3	(29.3–31.2)	16368
Family Health Coverage^f^			
no	68.7	(67.6–69.8)	42051
yes	31.3	(30.2–32.4)	18151
Total			60202

^a^Sample of subjects with 18+ years old that answered the ‘Pesquisa Nacional de Saúde’ individual questionnaire in 2013

^b^Prevalence (%), considering the complex sample design

^c^95 % Confidence Interval (CI), considering the complex sample design

^d^The categories of skin color are defined by the IBGE (Instituto Brasileiro de Geografia e Estatística) and reported by the respondent. IBGE 2010

^e^Population covered by the Brazilian Public Health System, or SUS (in Portuguese)

^f^“Family Health” refers to “Equipe de Saúde da Família”; a community based primary health care program funded by the Brazilian Public Health System or SUS

Among men, around 35 % had not visited a physician and 60 % had not visited a dentist in the last year; among women, these proportions were 18 and 52 %, respectively. Approximately two in ten individuals had not had their blood pressure checked in the last year and about 42 % had not had their blood glucose tested. Regarding utilization outcomes 0.7 % of the sample had never visited a physician, 3.3 % had never visited a dentist, 3 % had never had their blood pressure checked, and 11.5 % had never had their blood glucose tested (Table 2).

**Table 2:** Prevalence of healthcare utilization, by type of health service and sex. Brazilian National Health Survey, 2013^a^

	Male (*n* = 25,920) % (95 % CI)^b^	Female (*n* = 34,282) % (95 % CI)^b^	Total (*n* = 60,202) % (95 % CI)^b^
Last physician visit			
<12 months	65.5 (64.4–66.6)	82.0 (81.2–82.7)	74.2 (73.5–74.9)
>=1 year and <2 years	14.6 (13.9–15.4)	10.1 (9.6–10.7)	12.2 (11.8–12.7)
>=2 years and <3 years	6.1 (5.6–6.7)	3.1 (2.8–3.5)	4.5 (4.2–4.8)
>=3 years	12.7 (11.9–13.4)	4.5 (4.1–4.9)	8.3 (7.9–8.8)
Never	1.1 (0.9–1.4)	0.3 (0.2–0.5)	0.7 (0.6–0.8)
Last dentist visit			
<12 months	40.6 (39.4–41.8)	47.8 (46.8–48.9)	44.4 (43.6–45.2)
>=1 year and <2 years	19.3 (18.4–20.1)	19.3 (18.5–20.0)	19.3 (18.7–19.8)
>=2 years and <3 years	9.7 (9.1–10.3)	8.2 (7.7–8.7)	8.9 (8.5–9.3)
>=3 years	26.1 (25.1–27.1)	22.3 (21.5–23.1)	24.1 (23.4–24.8)
Never	4.4 (4.0–4.8)	2.4 (2.1–2.7)	3.3 (3.1–3.6)
Last blood pressure check			
<6 months	62.6 (61.5–63.6)	72.4 (71.5–73.3)	67.8 (67.1–68.5)
>= 6 months and <1 year	13.4 (12.7–14.2)	12.5 (11.9–13.2)	12.9 (12.4–13.5)
>=1 year and <2 years	9.5 (9.0–10.2)	7.6 (7.2–8.1)	8.5 (8.2–8.9)
>=2 years and <3 years	3.5 (3.1–3.9)	2.2 (2.0–2.5)	2.8 (2.6–3.0)
>=3 years	6.6 (6.1–7.2)	3.5 (3.2–3.9)	5.0 (4.7–5.3)
Never	4.3 (3.9–4.8)	1.7 (1.5–2.0)	3.0 (2.7–3.2)
Last blood glucose check			
<6 months	34.3 (33.2–35.3)	45.6 (44.6–46.6)	40.3 (39.5–41.1)
>= 6 months and <1 year	16.2 (15.4–17.1)	18.1 (17.4–18.9)	17.2 (16.7–17.8)
>=1 year and <2 years	15.1 (14.3–15.9)	15.3 (14.6–15.9)	15.2 (14.7–15.7)
>=2 years and <3 years	6.3 (5.8–6.8)	5.0 (4.6–5.4)	5.6 (5.3–5.9)
>=3 years	12.4 (11.7–13.1)	8.2 (7.7–8.8)	10.2 (9.7–10.6)
Never	15.8 (14.9–16.7)	7.8 (7.3–8.3)	11.5 (11.0–12.1)
Total	100.0	100.0	100.0

^a^Sample of subjects with 18+ years old that answered the ‘Pesquisa Nacional de Saúde’ individual questionnaire in 2013

^b^Prevalence (%) and 95 % Confidence Interval (CI), considering the complex sample design

Table 3 shows that 15 % of respondents underutilized the health system, representing approximately 22 million people aged 18 or older. The proportion of those excluded is highest among young people (those aged 18 to 29 = 24 %), men (20 %), non-white persons (19 %), those with no schooling or incomplete secondary schooling (19 %), and those without private health insurance (19 %). It is notable that almost half of those in class “E” and 24 % of those in class “D” underutilized the health system (Table 3).

**Table 3:** Prevalence of healthcare underutilization, according to socioeconomic characteristics and health care coverage. Brazilian National Health Survey, 2013^a^

Variable/categories	Underutilization^b^ %^c^	95 % CI^d^
Sex		
Male	20.3	(19.4–21.3)
Female	10.4	(9.8–11.1)
Skin Color^e^		
White	10.9	(10.2–11.7)
Non-white	18.9	(18.1–19.7)
Educational level		
No education/primary incomplete	19.5	(18.6–20.5)
Secondary incomplete	19.4	(18.0–21.0)
Undergraduate (college) incomplete	12.2	(11.3–13.0)
Undergraduate complete and above	3.8	(3.1–4.5)
Social Class		
E	44.2	(40.6–47.9)
D	24.4	(23.1–25.8)
C	14.7	(13.9–15.5)
B	7.3	(6.6–8.2)
A	2.8	(1.8–4.3)
Age (years)		
>=18 and <30	24.5	(23.2–25.9)
>=30 and <40	15.2	(14.1–16.3)
>=40 and <50	12.0	(11.0–13.0)
>=50 and <60	9.6	(8.5–10.9)
>=60	9.4	(8.5–10.5)
Private Health Insurance		
No^f^	19.2	(18.5–20.0)
Yes	5.6	(5.0–6.3)
Family Health Care Coverage^g^		
No	13.7	(13.0–14.4)
Yes	18.1	(17.1–19.2)
Total	15.1	(14.5–15.7)

^a^Sample of subjects with 18+ years old that answered the ‘Pesquisa Nacional de Saúde’ individual questionnaire in 2013

^b^Composed of individuals that fulfilled one or more of these conditions: never visited a physician; or never visited a dentist; or never checked the blood glucose; or never checked the blood pressure

^c^Prevalence (%), considering the complex sample design

^d^95 % Confidence Interval (CI), considering the complex sample design

^e^The categories of skin color are defined by the IBGE (Instituto Brasileiro de Geografia e Estatística) and reported by the respondent. IBGE 2010

^f^Population covered by the Brazilian Public Health System, or SUS (in Portuguese)

^g^“Family Health” refers to “Equipe de Saúde da Família”; a community based primary health care program funded by the Brazilian Public Health System or SUS

The results of the logistic regression model are presented in Table 4. With exception of Family Health Strategy coverage, all others covariates were significantly associated with underutilization. The multivariate model shows a higher likelihood of underutilization of the health system among individuals of economic class “E” (AOR = 6.31, 95 % CI = 3.76–10.61), younger adults (AOR = 4.40, 95 % CI = 3.78–5.12), those with no schooling or incomplete primary schooling (AOR = 2.93, 95 % CI = 2.30–3.74), males (AOR = 2.16, 95 % CI = 1.99–2.35), and those without private health insurance (AOR = 2.11, 95 % CI = 1.83–2.44). With respect to race, individuals self-classified as “white” were less likely to report underutilization (AOR = 0.82, 95 % CI = 0.75–0.90).

**Table 4:** Factors associated with underutilization of health care^a^. Brazilian National Health Survey, 2013^b^

Variable/categories	AOR^c^	95 % CI^d^
Sex		
Male	2.16	(1.99–2.35)
Female	1.00	–
Skin Color^e^		
White	0.82	(0.75–0.90)
Non-white	1.00	–
Educational level		
No education/primary incomplete	2.93	(2.30–3.74)
Secondary incomplete	2.35	(1.84–3.00)
Undergraduate (college) incomplete	1.72	(1.37–2.14)
Undergraduate complete and above	1.00	–
Social Class		
E	6.31	(3.76–10.61)
D	3.03	(1.84–4.99)
C	1.84	(1.27–3.01)
B	1.36	(0.84–2.02)
A	1.00	–
Age (years)		
>=18 and <30	4.40	(3.78–5.12)
>=30 and <40	2.44	(2.10–2.83)
>=40 and <50	1.66	(1.43–1.94)
>=50 and <60	1.17	(0.98–1.41)
>=60	1.00	–
Private Health Insurance		
No^f^	2.11	(1.83–2.44)
Yes	1.00	–
Family Health Care Coverage^g^		
No	0.97	(0.88–1.07)
Yes	1.00	–

^a^Variable composed of individuals that fulfilled one or more of these conditions: never visited a physician; or never visited a dentist; or never checked the blood glucose; or never checked the blood pressure

^b^Sample of subjects with 18+ years old that answered the ‘PesquisaNacional de Saúde’ individual questionnaire in 2013

^c^Adjusted Odds Ratio (AOR) of underutilizing the health care system, considering the complex sample design

^d^95 % Confidence Interval (CI) of the Adjusted Odds Ratio (AOR) of underutilizing the health care system, considering the complex sample design

^e^The categories of skin color are defined by the IBGE (‘Instituto Brasileiro de Geografia e Estatística) and reported by the respondent.IBGE 2010

^f^Population covered by the Brazilian Public Health System, or SUS (in Portuguese)

^g^“Family Health” refers to “Equipe de Saúde da Família”; a community-based primary health care program funded by the Brazilian Public Health System or SUS

## Discussion

Almost 22 million Brazilians underutilized their healthcare system in 2013, representing 15 % of the adult population. The findings from this study demonstrate a situation in which the poor, those with lower educational attainment, non-whites, and males had a higher likelihood of never accessing one or more of these services: a physician or dentist consultation, or blood glucose or blood pressure screening. A study by Macinko and Lima-Costa [3] employed data from previous national household sample surveys (Pesquisa Nacional por Amostra de Domicílios, or PNAD in Portuguese) from 1998 to 2008 and found evidence that inequity in healthcare utilization, measured by the probability of a medical, dental, or hospital visit during the previous 12 months, decreased over the sampling period, but that the services remained concentrated among the richest. Compared with our study, which examined the outcomes of not visiting a doctor or dentist, or not undergoing basic health screening, the unfavorable gradient among the poorest was more than six times higher than among the richest, indicating that despite reduction in inequities, utilization disparities persisted in the Brazilian health system. These findings were similar to a previous study conducted in 2003 using data from a nationally representative survey [12].

Another study employing the PNAD also found decreased social disparities in healthcare utilization between 1998 and 2005, measured by the utilization of health services in the previous 15 days. The likelihood of not being cared for by the health system was higher among males, people with lower educational attainment, and among the poorest [13]. The factors associated with healthcare utilization in this previous study were similar to those in our study, with minor differences in the magnitude of the gap [13]. The increasing coverage of community-based health workers provided by the SUS may be one of the factors responsible for the decline in healthcare inequities in Brazil. The portion of the population served by community health workers increased from 29.6 % in 1998 to 60.4 % in 2008, and that served by family health teams increased from 6.6 to 49.5 % over the same period [12]. This suggests that it is possible, through modification of the health system, to improve access and change utilization patterns.

According to Penchansky and Thomas [14], the concept of “access” goes beyond the use of the health system, and has five dimensions: availability, accessibility, accommodation, affordability, and acceptability. Levesque et al. [15] defines “access” as “the interface between potential users and healthcare resources, influenced by the characteristics of those who supply as well as those who utilize the services.” In Levesque’s formulation, there are five “abilities” attributed to individuals who interact with services: the ability to perceive, to seek, to reach, to pay (for the services), and the ability to engage [15]. In other words, healthcare utilization is determined through the interaction between individual characteristics and healthcare system attributes. To illustrate this concept with our findings, we can look at the example of gender and healthcare utilization. Historically, women utilize the health system more frequently than men [16], as was seen in our study, indicating that population characteristics (in this case, gender) can determine healthcare utilization patterns.

Lavesque’s [15] framework indicates our study’s main limitation, namely the difficulty of differentiating whether the outcome was a result of healthcare system attributes or individual characteristics. Another issue related to our outcome is the differentiation among healthcare access and healthcare quality, since it is possible for an individual to be attended by a healthcare worker and not receive blood glucose or blood pressure screening. In this case it was impossible to differentiate, using the PNS data, if the patient was not screened at all (due lack of access or unavailability of this service) or was screened but did not receive the results (low quality of the services). In both cases we classified this outcome as underutilization of services. The magnitude of our results, which suggest underutilization of the health system by the most vulnerable among the population and by SUS-dependent citizens, speaks to the gravity of the situation.

Another limitation is the possibility of recall bias. A randomized study conducted in Ghana observed lower self-reported healthcare utilization prevalence compared to a diary provided to individuals [17] and another study identified recall bias for healthcare utilization among the older population [18]. It is also possible that lower socioeconomic status might be related to recall bias. Thus, it is reasonable to assume that our results might be over-estimated. However, results from the final model show that people aged 60 years or more are less likely to underutilize healthcare suggests the opposite.

This study did not analyze subjects that responded they had “never used” or “never had” to more than one of the four possible the healthcare questions, but such an analysis could help to clarify the conditionality of a prior doctor visit on access to screening exams and to assess people’s “degree of underutilization” (people reporting “no” to 2, 3 or four questions). These analyses could be conducted in future publications, since 2.9 % of the population reported underutilization for two or more of the four possible items (data not shown).

Despite the universal coverage of the Brazilian unified health system, 27.9 % of the population had private healthcare in 2013 [19]. This scenario explains the almost-universal access to physicians, but also highlights an intriguing disparity. The likelihood of healthcare utilization is two-times higher among the population that uses the private healthcare sector, possibly due to a higher demand for preventive care in this group [20]. Lower utilization among SUS users could also be related to barriers to access, unequal supply of health services, or even a lack of information regarding preventive services [20]. Another barrier might be related to the distribution of healthcare facilities in Brazil: despite the fact that Southern regions had a higher concentration of healthcare facilities, social inequalities in healthcare access are higher in magnitude there than other regions, indicating some inter- and intra-regional variation [21].

A study comparing private versus public (SUS) healthcare users in Brazil found that the most prevalent motivation for seeking healthcare was illness followed by preventive care, in both populations. However, the proportion of individuals seeking care due to illness was higher among SUS users (58,4 % vs. 40.9 %), and the proportion of individuals seeking services for preventive care was higher among users of private healthcare (26.1 % vs.23.5 %), with both results being statistically significant [22].

With respect to the availability of services, an analysis of our data (not shown in results) reveals that among the 15.4 % of individuals who sought a health consultation in the previous 2 weeks, 4.7 % were not seen, and, of those, 43.0 % were not attended to due to the lack of a physician, 36.2 % due to insufficient appointments, and only 7.1 % due to lack of specialized staff. These results indicate some degree of unavailability of health services, but relatively low when compared to other health systems, like in the UK, where 11 % of the population who sought an appointment in primary health care facilities were able to get it in the first time they try [23]. In USA these numbers are even lower, and the frequency of not getting an appointment for the first time in primary care ranged from 15.3 % among privately insured patients, 42.1 % for medicaid and 84.6 % for uninsured patients that were able to pay U$75 or less in cash during their first visit [24].

The most prevalent outcome of underutilization was blood glucose screening, with more than one-tenth of the study population reporting that they had never undergone this test. This result represents a serious issue, because diabetes represents the fourth most prevalent non-communicable disease [5], and the third in terms of lost DALYs, among the Brazilian population [6]. This problem is complex, considering that the prevalence of self-reported diabetes is lower among those who did not undergo blood glucose screening [25]. Since the prevalence of individuals who never went to a physician is relatively low (less than 1 %), we can assume that the lack of blood glucose screening is a problem related to barriers to laboratory services, or to under-ordering of this test by physicians. Since primary healthcare facilities within SUS have well-established protocols for ordering health screening exams, such as blood glucose and blood pressure [5], this finding supports the hypothesis that barriers to laboratory services may explain the disparity in health screening we observed in our sample.

Considering previous national studies, 18.7 % of the Brazilian population reported having never visited a dentist in 1998, with a downward trend in subsequent years: 15.9 % in 2003 and 11.6 % in 2008 [26]. Our results show that only 3.3 % did not visit a dentist in 2013, further supporting a potential downward trend (not tested) associated with the implementation of the National Oral Health Policy (*Brasil Sorridente*, in Portuguese) which expanded access to dental care across the country [1]. The likelihood of never having visited a dentist in 1998 was 16 % higher among people in the poorest quintile compared to the richest, considering adults aged 20–49, and 7.6 times higher among the poorest versus the richest, considering people of all age groups [27]. Although not tested, this result suggests evidence of improvement in access to this type of treatment. Nevertheless, 70.6 % of the Brazilian population sought dental treatment in private clinics (data not shown), indicating a relatively low coverage in public health system and explaining partially the gradient of underutilization toward the poorest.

## Conclusion

In conclusion, considering the Brazilian context of free universal and equal healthcare guaranteed by the National Constitution, the recent expansion of primary healthcare and oral health programs, and recent reductions in healthcare inequities, we still observe a significant gap in utilization of healthcare with underutilization, specially the lack of blood glucose screening, concentrated among the most vulnerable in the Brazilian population. Access to physicians was virtually universal, but visiting a dentist and having blood pressure measured are still problems. We recommend further projects both promoting awareness of healthcare utilization and improving the coverage of laboratory services in order to bolster universal and equalitarian healthcare in Brazil. Health communication is also a possible way to empower the citizens, improving the awareness of healthcare utilization and the relationship between users and health services.
